# Author Correction: Streamlined structure determination by cryo-electron tomography and subtomogram averaging using TomoBEAR

**DOI:** 10.1038/s41467-024-49476-7

**Published:** 2024-07-03

**Authors:** Nikita Balyschew, Artsemi Yushkevich, Vasilii Mikirtumov, Ricardo M. Sanchez, Thiemo Sprink, Mikhail Kudryashev

**Affiliations:** 1 Max Planck Institute of Biophysics, Frankfurt on Main, Germany; 2 Buchmann Institute for Molecular Life Sciences, Goethe University of Frankfurt on Main, Frankfurt, Germany; 3 In Situ Structural Biology, Max Delbrück Center for Molecular Medicine in the Helmholtz Association, Berlin, Germany; 4 Department of Physics, Humboldt University of Berlin, Berlin, Germany; 5 Institute of Chemistry and Biochemistry, Freie Universität Berlin, Takustraße 6, Berlin, Germany; 6 Core Facility for Cryo-Electron Microscopy, Charité-Universitätsmedizin Berlin, Berlin, Germany; 7 Cryo-EM Facility, Max Delbrück Center for Molecular Medicine in the Helmholtz Association, Berlin, Germany; 8 Institute of Medical Physics and Biophysics, Charité-Universitätsmedizin Berlin, Berlin, Germany; 9 Present Address: EMBL Heidelberg, Heidelberg, Germany

**Keywords:** Cryoelectron tomography, Software, Biochemistry, Biophysics

Correction to: *Nature Communications* 10.1038/s41467-023-42085-w, published online 17 October 2023

The original version of this Article contained an error in the author affiliations.

The affiliation of Vasilii Mikirtumov with Institute of Chemistry and Biochemistry, Freie Universität Berlin, Takustraße 6, Berlin, Germany was inadvertently omitted.

