# Supplementary material for: Streamlined structure determination by cryo-electron tomography and subtomogram averaging using TomoBEAR
Source: Nat Commun. 2023 Oct 17;14:6543. doi: 10.1038/s41467-023-42085-w (PMC10582028; doi:10.1038/s41467-023-42085-w)
Supplement: Supplementary file 2 — Supplementary Information [file 41467_2023_42085_MOESM2_ESM.pdf]

Supplementary information

Streamlined Structure Determination by Cryo-Electron Tomography and Subtomogram Averaging using TomoBEAR

Nikita Balyschew[1,2,$], Artsemi Yushkevich[3,4,$], Vasilii Mikirtumov[1,3], Ricardo M. Sanchez[1,2,8], Thiemo Sprink[5,7], Misha Kudryashev[1,2,3,6]*

1. Max Planck Institute of Biophysics, Frankfurt on Main, Germany.
2. Buchmann Institute for Molecular Life Sciences, Goethe University of Frankfurt on Main, Germany.
3. *In Situ* Structural Biology, Max Delbrück Center for Molecular Medicine in the Helmholtz Association, Berlin, Germany.
4. Department of Physics, Humboldt University of Berlin, Germany
5. Core Facility for Cryo-Electron Microscopy, Charité-Universitätsmedizin Berlin, Germany.
6. Institute of Medical Physics and Biophysics, Charité-Universitätsmedizin Berlin, Germany.
7. Cryo-EM Facility, Max Delbrück Center for Molecular Medicine in the Helmholtz Association, Berlin, Germany.
8. Present address: EMBL Heidelberg, Germany.

$ indicates equal contribution

*Email: mikhail.kudryashev@mdc-berlin.de

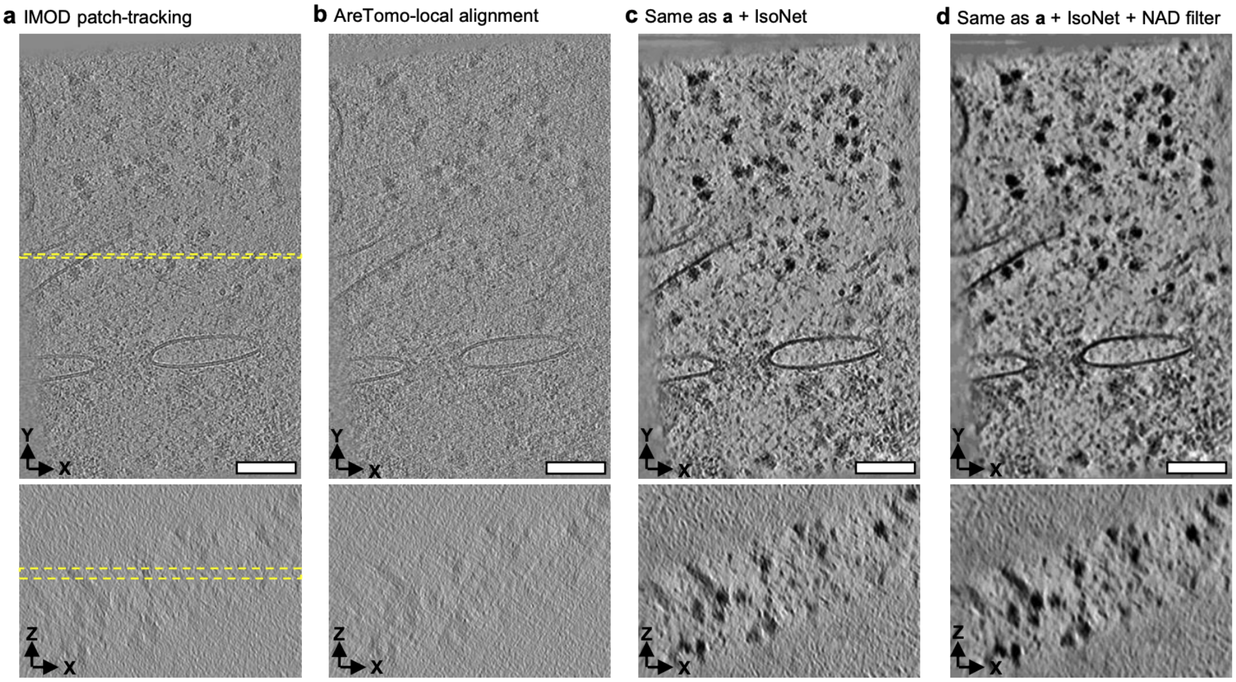

Supplementary Fig. 1. TomoBEAR functionality for fiducial-less alignment and post-processing of tomograms. All panels represent the same XY (top row) and XZ (bottom row) slices of the same example tomogram reconstructed by the weighted back projection algorithm from IMOD from the EMPIAR-11306 benchmarking data set (*in situ* pFIB-milled lamellae): A. Reconstructed from tilt stack aligned by patch-tracking in IMOD. B. Reconstructed from tilt stack locally aligned by AreTomo. C. Reconstructed from the same aligned tilt stack as in A, but with additional CTF deconvolution, denoising and missing wedge restoration performed by IsoNet. D. Reconstructed from the same aligned tilt stack as in A, and post-processed as in C, but with the additional non-anisotropic diffusion (NAD) filter applied by IMOD. The scale bar on all images is 100 nm. The yellow dashed rectangles on A represent the location of a section in the XZ and XY slices, which were summed and presented as the corresponding images A-D.

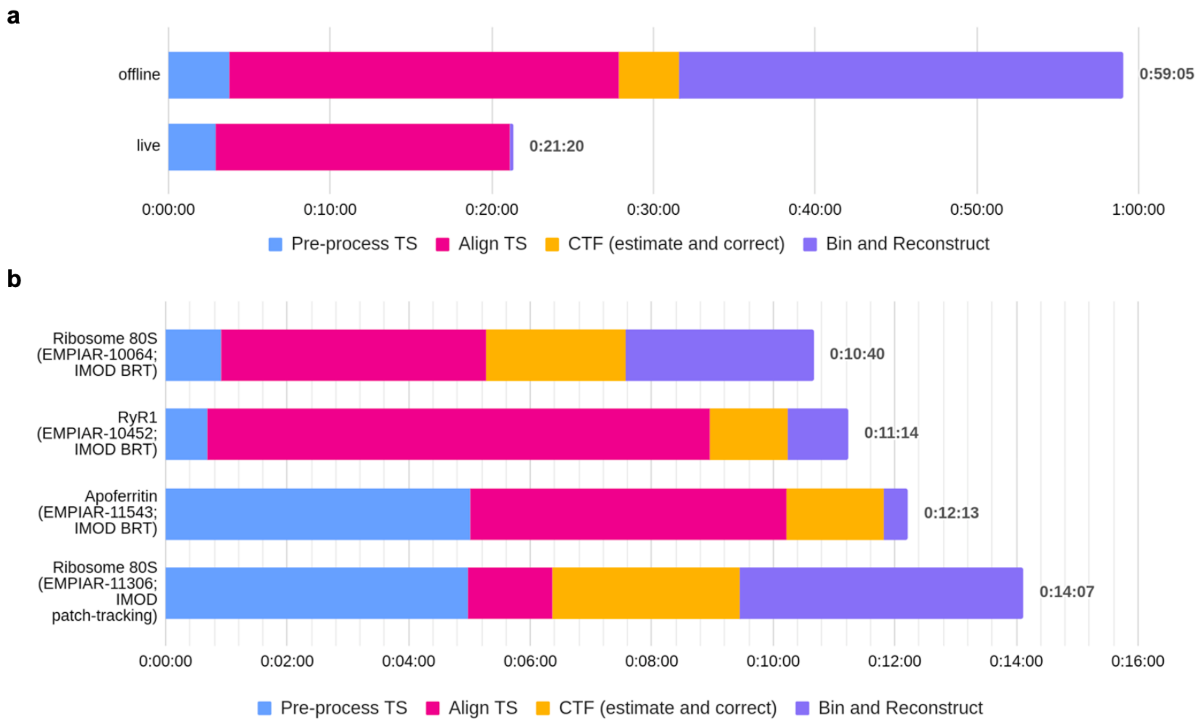

Supplementary Fig. 2. Benchmarking TomoBEAR performance for tomographic reconstruction. A. Processing times of the HIV-1 data set (EMPIAR-10164) for offline (conventional) and live data processing modes. B. Processing times for tomographic reconstruction of the four processed data sets in the conventional data processing mode. The provided time is per-tomogram and calculated by averaging processing time was measured for four tomograms. Time format on both panels: hh:mm:ss.SS, where hh - hours, mm - minutes, ss - seconds.

The processing stages in Supplementary Fig. 2 included the following operations and TomoBEAR modules:

- Pre-process TS - harvesting metadata (MetaData), sorting of tilt movies (SortFiles), motion correction (MotionCor2), create raw stacks (CreateStacks);
- Align TS - pre-aligned stack generation (BatchRunTomo), fiducials tracking and fiducials-based alignment (BatchRunTomo, DynamoTiltSeriesAlignment, DynamoCleanStacks) or patched (fiducial-less) alignment (AreTomo, BatchRunTomo);
- CTF (estimate and correct) - CTF estimation and correction (GCTFCtfphaseflipCTFCorrection, BatchRunTomo);
- Bin and Reconstruct - stacks binning (BinStacks) and tomographic reconstruction (Reconstruct).

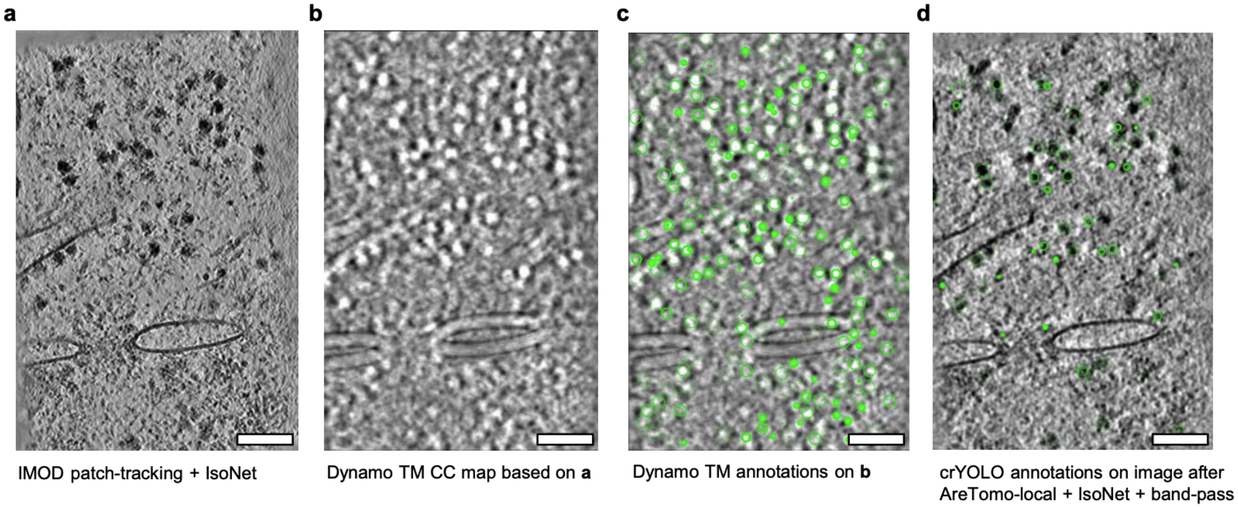

Supplementary Fig. 3. TomoBEAR functionality for particle picking of 80S ribosomes. All panels represent the same XY slice based on the same example tomogram reconstructed by the weighted back projection algorithm from IMOD from the EMPIAR-11306 benchmarking data set (pFIB-milled lamellae). A. Slice of the tomogram reconstructed from the tilt stack aligned by patch-tracking in IMOD. Image contrast was enhanced for representation purposes by CTF deconvolution, denoising and missing wedge restoration using IsoNet. B. Slice of the cross-correlation (CC) map as the result of the Dynamo-based template matching, performed on the tomogram aligned as in A. C. The same image as in B, but annotated (green circles) with corresponding particle positions identified by template matching post-processing routines of TomoBEAR. D. Reconstructed from tilt stack locally aligned by AreTomo, with additional denoising and missing wedge restoration performed by IsoNet as well as band-pass filtering as pre-processing step of crYOLO. C. The same image as in D, but annotated (green circles) with corresponding particle positions identified by crYOLO training and prediction functionality. The scale bar on all images is 100 nm.

Supplementary Text 1: Description of TomoBEAR modules and their dependencies

Generally, all the modules can be subdivided into the following categories:

- Global parameters configuration: general;
- Pipeline control: StopPipeline;
- Tilt-series and cryo-ET data processing: MetaData, SortFiles, MotionCor2, GridEdgeEraser, CreateStacks, DynamoTiltSeriesAlignment, DynamoCleanStacks, AreTomo, BatchRunTomo, GCTFCtfphaseflipCTFCorrection, BinStacks, Reconstruct, IsoNet;
- Particles picking-associated modules: DynamoImportTomograms, EMDTemplateGeneration, TemplateGenerationFromFile, DynamoTemplateMatching, TemplateMatchingPostProcessing, crYOLO, GenerateParticles;
- subtomogram averaging modules: DynamoAlignmentProject.

The modules functional is described below, followed by the dependencies table and tested dependencies versions.

The continuously updated version of the information presented in this supplementary material can be found on the following TomoBEAR documentation page:
https://github.com/KudryashevLab/TomoBEAR/wiki/Modules

General

The *general* section is not a module, but a section where all the general parameters regarding the processing and the environment can be found. Parameters which are input in this section are visible to all modules during the execution. If parameters with the same key are found in a module block, then they override parameters from the *general* section.

MetaData

The *MetaData* module collects descriptive statistics such as min, max, mean, std from the raw data.

SortFiles

The *SortFiles* module sorts the raw files into tilt-series corresponding to tomograms and links them to their corresponding folders for further processing.

MotionCor2

The *MotionCor2* module uses MotionCor2[1] to correct for the sample movement in a given projection recorded as a dose-fractionated movie or EER sequence. For the options it is advised to look also into the manual of MotionCor2 provided along with the source code as there are some more detailed descriptions.

GridEdgeEraser
The GridEdgeEraser module contains an original algorithm that performs grid edge identification in the raw tilt stacks and erases it for Au grids data. The algorithm is based on the cross-correlation iterative roto-translational alignment and image intensity statistics.

CreateStacks
The *CreateStacks* module creates the stacks and normalizes them. There are two options for normalization, the default scheme is to divide the projections by their frame count. TomoBEAR detects automatically if you are using high-dose images from hybrid StA[2] and divides them by their corresponding frame count in contrast to low-dose images where the frame-count is different.

DynamoTiltSeriesAlignment
The *DynamoTiltSeriesAlignment* module uses the tilt stacks alignment algorithm from Dynamo[3] which is the state of the art available algorithm for fiducial-based alignment. As default, reasonable parameters for many cryo-ET projects are set. Some of them are dynamically derived. The option to override non-dynamically derived parameters is available via the JSON configuration file. For troubleshooting and optimization of parameters it is possible to go to the processing folder and use the Dynamo tools as in the tutorial (https://wiki.dynamo.biozentrum.unibas.ch/w/index.php/Walkthrough_on_GUI_based_tilt_series_alignment).
DynamoCleanStacks
The *DynamoCleanStacks* module can be run after the *DynamoTiltSeriesAlignment* to automatically clean up the tilt stacks and to remove the projections where *DynamoTiltSeriesAlignment* has not found gold beads. For that *DynamoCleanStacks* uses the output from Dynamo tilt stacks alignment which states on which projections the fiducials could be fit. The others are then removed from the tilt stacks for further processing.

AreTomo
The AreTomo module uses AreTomo[4] to perform global or local fiducial-free alignment of the tilt stack. In order to optimize alignment parameters, please consult with the corresponding AreTomo documentation.

StopPipeline
The *StopPipeline* module allows to stop TomoBEAR after some processing step to inspect the output and not waste computational resources if parameters need to be optimized.

BatchRunTomo
The *BatchRunTomo* is a versatile module performing IMOD operations[5]. The detailed description can be found here: Tomography Guide for IMOD Version 4.11. TomoBEAR can runs steps of batchruntomo:
0: Setup
1: Preprocessing
2: Cross-correlation alignment

3: Pre Aligned stack
4: Patch tracking, auto seeding, or RAPTOR
5: Bead tracking
6: Alignment
7: Positioning
8: Aligned stack generation
9: CTF plotting
10: 3D gold detection
11: CTF correction
12: Gold erasing after transforming fiducial model or projecting 3D model
13: 2D filtering
14: Reconstruction
14.5: Postprocessing on a/b axis reconstruction
15: Combine setup
16: Solvematch
17: Initial matchvol;
18: Autopatchfit
19: Volcombine
20: Post Processing with Trimvol
21: NAD (Nonlinear anisotropic diffusion)

GCTFCtfphaseflipCTFCorrection
The *GCTFCtfphaseflipCTFCorrection* module is detecting the defocus using GCTF[6] or CTFFIND4[7] on the tilt series which will be further CTF-corrected and used to reconstruct tomograms for template matching or particle generation. The results can be examined in the processing folders. As well, the module can be used for CTF-correction using Ctfphaseflip from IMOD.

BinStacks
The *BinStacks* module is used to bin tilt series stacks to be able to reconstruct them with the *Reconstruct* module. Stacks with selected binning levels will be produced.

Reconstruct
The *Reconstruct* module is used for tomogram reconstruction from aligned tilt stacks . The module is set up by default to reconstruct CTF-corrected binned stacks. If you otherwise want to reconstruct unbinned stacks or non-CTF-corrected stacks (for example, for further 3D CTF-deconvolution by IsoNet) you need to set up the *Reconstruct* module properly. Besides, there are several post-filtering options available in the module such as exact filtering and NAD filtering by IMOD.

IsoNet
The IsoNet module provides functionality of the deep learning framework IsoNet[8] used for reconstruction of the missing wedge region in tomograms. Besides, IsoNet provides an interface for 3D CTF-deconvolution and denoising. TomoBEAR interface includes such IsoNet routines as pre-processing (STAR file preparation, mask creation, CTF-deconvolution), training (refinement)

and prediction. To optimize parameters of the software please use the corresponding IsoNet documentation (https://isonetcryoet.com/).

DynamoImportTomograms

The *DynamoImportTomograms* module generates a Dynamo Catalogue for the user and inputs the tomograms to that catalogue. After that you can call the Dynamo Catalogue Manager (dcm) to generate the models for the tomograms or pick particles in them using the functionality of Dynamo Catalogue[9].

EMDTemplateGeneration

The *EMDTemplateGeneration* module is used to automatically download a EMDB (https://www.ebi.ac.uk/emdb/) template map which is further down-scaled to match the requested template matching binning level. Besides that, an automated routine to generate the mask is also implemented. Either this module or the module TemplateGenerationFromFile needs to be run before template matching is executed by the DynamoTemplateMatching module.

TemplateGenerationFromFile

The *TemplateGenerationFromFile* module imports a map into the TomoBEAR workflow given by a path and scales it properly if the map header contains the correct pixel size; else the pixel size can be input as a parameter through the JSON-based configuration file. Either this module or the module EMDTemplateGeneration needs to be run before template matching is executed by the DynamoTemplateMatching module.

DynamoTemplateMatching

The *DynamoTemplateMatching* module re-implements the template matching functionality from the Dynamo package, but distributes calculations on the GPU. The GPU-distributed version executes is up to 12-15 times faster than the conventional CPU-distributed template matching implementation available in Dynamo[3].

TemplateMatchingPostProcessing

The *TemplateMatchingPostProcessing* module takes the cross-correlation volumes generated by the *DynamoTemplateMatching* module and extracts the coordinates from the peaks found in them until a given threshold is reached. This threshold is set by default to a value of 2.5 standard deviations. Furthermore, this module could extract the corresponding subtomograms by cropping them from the reconstructed tomograms using Dynamo or reconstruct them directly from the aligned stacks using SUSAN.

crYOLO

The crYOLO module provides functionality of the deep learning framework crYOLO[10] used for particle coordinate prediction. TomoBEAR interface includes the crYOLO routines: pre-processing (configuration file preparation, low-pass or neural network-based filtering), training and prediction. To optimize parameters of the training and prediction please use the corresponding crYOLO documentation (https://cryolo.readthedocs.io/en/stable/index.html).

GenerateParticles

The GenerateParticles module uses Dynamo-like particles tables to extract particles in one of the following ways:

- crop sub-tomograms from the reconstructed tomograms using Dynamo;
- reconstruct sub-tomograms from sub-stacks cropped directly from aligned tilt stacks using SUSAN.

DynamoAlignmentProject

The *DynamoAlignmentProject* module can be set up to generate the two common Dynamo[3] subtomogram averaging projects: multiple reference alignment (MRA) project and an independent half-set based refinement project. For classification we find it useful to have one or two classes with the correct reference that was used e.g. for template matching and some classes containing only noise ("noise traps") which will attract suboptimal particles into classes for removal.

Modules dependencies

The only mandatory dependency of any TomoBEAR module is the MATLAB or its pre-compiled libraries. The three other essential dependencies of the TomoBEAR code base are:

- CUDA, which is utilized by many interfaced software packages used by TomoBEAR;
- IMOD and Dynamo, with both of them met in approximately half of the modules being used there as main module functionality or as auxiliary tool sets.

All the dependencies, except for mandatory one, corresponding to the modules which depend on them are listed in the table below (optional dependencies are provided in the brackets):

Module	Dependencies
MetaData	no additional dependencies
SortFiles	no additional dependencies
MotionCor2	IMOD, MotionCor2, CUDA
GridEdgeEraser	Dynamo
CreateStacks	IMOD, Dynamo
DynamoTiltSeriesAlignment	Dynamo
DynamoCleanStacks	IMOD, Dynamo
AreTomo	IMOD, AreTomo, CUDA
BatchRunTomo	IMOD
GCTFCtfphaseflipCTFCorrection	IMOD, Gctf/CTFFIND4, CUDA
BinStacks	IMOD

Module	Dependencies
Reconstruct	IMOD
IsoNet	(IMOD), IsoNet, (Anaconda), CUDA
DynamoImportTomograms	Dynamo
EMDTemplateGeneration	Dynamo
TemplateGenerationFromFile	Dynamo
DynamoTemplateMatching	Dynamo, (CUDA)
TemplateMatchingPostProcessing	Dynamo, (SUSAN), (CUDA)
crYOLO	(Dynamo), crYOLO, (Anaconda), CUDA
GenerateParticles	Dynamo, (SUSAN), (CUDA)
DynamoAlignmentProject	Dynamo, (SUSAN), (CUDA)

Further we provide a list of the dependency's versions, which were used for TomoBEAR testing:

- MATLAB: 2021a/b;
- CUDA: 10.1 or/and 11.5 (depends on the certain software requirement);
- IMOD: 4.11.24 (compiled by its authors on RHEL7 with CUDA-10.0 and Qt5 and used by us with CUDA-10.1), 4.9.12 (compiled by its authors on RHEL7 with CUDA-8.0 and Qt4 and used by us with CUDA-10.1);
- Dynamo: 1.1.532 with MRC-9.9.0 for GLNXA64 system (with GPU-based functionality compiled by us locally using CUDA-11.5);
- MotionCor2: 1.4.4 (pre-compiled by its authors for CUDA-11.3 which was executed by us using CUDA-11.5);
- AreTomo: 1.3.3 (pre-compiled by its authors and used by us with CUDA-11.5);
- Gctf: v1.18 (pre-compiled by its authors and used by us with CUDA-10.1);
- CTFFIND4: 4.1.14 (released on May 8, 2020);
- IsoNet: 0.2 (using CUDA-11.5);
- crYOLO: 1.9.3 (using CUDA-11.5);
- SUSAN: v0.1-RC1-TomoBEAR (compiled by us using CUDA-11.5).

Specifically, for IsoNet and crYOLO we provide as well information on the core used libraries versions, we tested for the corresponding Anaconda environments:

- IsoNet libraries: python=3.9, cudatoolkit=11.5, cudnn=8.3, tensorflow-gpu=2.11;
- crYOLO libraries: python=3.8, nvidia-cudnn-cu115=8.3, nvidia-tensorflow=1.15.

Supplementary Text 2: Development of new TomoBEAR modules

Development of new TomoBEAR modules to support external cryoET packages requires a continuous effort for testing, updating and maintaining them and is hard to perform by a small academic team. However, the open-source status of the TomoBEAR project enables the academic cryo-ET community to overcome limits of a single team by contributing to the software accumulated broad community's experience and expertise. In the end, software is made for users' needs and the best way to deliver the requested functionality is to let users themselves participate in the development process. That is possible by means of open-source code distribution, open public communication channels and contribution guidelines, all of which are accessible for the TomoBEAR project via its GitHub repository page: https://github.com/KudryashevLab/TomoBEAR.
Here we provide a brief guide on how to write new TomoBEAR modules to further assist and encourage TomoBEAR users on independent development of functionality for their specialized needs. Depending on the certain computational tool characteristics as well as its role in the TomoBEAR workflow, there are many ways to develop a new module. Available cryo-ET software represents a variety of kinds of computational tools which:

- have different kind of interfaces like command line (CLI), graphical (GUI) or programmatic (API);
- are based on different languages (Python, C++, CUDA, MATLAB, Java, etc.);
- are distributed as standalone executables and/or open-source code;
- utilize sequential and/or parallel processing with CPU and/or GPU parallelization;
- etc.

However, in order to help the users to overcome the entry development barrier, we prepared two module design templates, which are available on the GitHub repository:

- "modules/GeneralModuleTemplate.m" - for a general-purpose module (wrapping original algorithm or CLI tool);
- "modules/DLToolModuleTemplate.m" - for a (Python-based) deep-learning tool-oriented module;

as well as

- "configurations/defaults_template.json" - a configuration file, containing templates of sections for the two module templates above.

General-purpose module template

In order to create a new TomoBEAR module to wrap your original algorithm or any external CLI tool we suggest to use our general-purpose module template which is located in the TomoBEAR source code folder by the following relative path: modules/GeneralModuleTemplate.m. As well,

in the source code folder you may find the configuration file template by the following relative path: configurations/defaults_template.json.
Please, copy the module template file in the same “modules/” folder and rename it using the wished title for the new module. Please, add at the end of the default’s configuration JSON file “configurations/defaults.json” as well the corresponding section from the template configurations file:

```
"GeneralModuleTemplate": {
    "execution_method": "once",
    "new_module_param_number": default_value,
    "new_module_param_string": "default_value",
    "new_module_params_list": [default_value1,...,default_valueN],
    "new_module_params_section": {
        ... // put here module params and their defaults
    }
}
```

The configuration parameters template above contains examples of new custom module-specific parameters introduced of different nature as numerical (float or integer) value, string, list of numerical/string values or structure section which may contain all the previous parameter types. Note the mandatory “execution_method” parameter, which can be set by default to one of the following values according to the module execution (parallelisation) scheme:

- “once” - a one-time execution of the module functional (e.g. used by metadata-collecting modules like *MetaData*, by data importing/exporting modules like *EMDTemplateGeneration*). However, be aware that this execution method is also used to execute modules with internal (in-module) parallelisation (e.g. when a wrapped algorithm uses its own parallelisation scheme like modules *GenerateParticles*, *crYOLO* and *IsoNet* do).
- “sequential” - a sequential multi-time independent execution of the module functional on the different data chunks like tilt series or tomograms, when parallelization is not desired. As well, this method is useful and the only possibility for debugging modules which by default use parallel execution methods like “parallel” or “in_order” described below.
- “parallel” - a simultaneous multi-time independent execution of the module functional on the different data chunks like tilt series or tomograms, serves for functional parallelized on CPU's (e.g. modules *SortFiles*, *DynamoCleanStacks*, *BinStacks*);
- “in_order” - a simultaneous multi-time independent execution of the module functional on the different data chunks like tilt series or tomograms, serves for functional parallelized on GPU's (e.g. modules *MotionCor2*, *AreTomo*, *Reconstruct*, *DynamoTemplateMatching*);

- “control” - the special execution method reserved only for operation of the *StopPipeline* control module, which you should not normally use for a new module development.

The general-purpose module is inherited from the abstract class *Module* (source code is located at “modules/Module.m”), includes a license note (with license copyright holders and years, license type and version) and has the high-level structure as in the following template:

```
%%%%%%%%%%%%%%%%%%%%%%%%%%%%%%%%%%%%%%%%%%%%%%%
% LICENSE NOTE
%%%%%%%%%%%%%%%%%%%%%%%%%%%%%%%%%%%%%%%%%%%%%%%
classdef GeneralModuleTemplate < Module
  methods
    %% 0. Module instance constructor
    function obj = GeneralModuleTemplate(configuration)
      obj@Module(configuration);
    end

    %% 1. Pre-execution module setup
    function obj = setUp(obj) … end

    %% 2. Module execution (main method)
    function obj = process(obj) … end

    %% N. Custom processing method
    function [outputs] = customMethod(obj, inputs) … end

    %% 3. Post-execution routines
    function obj = cleanUp(obj) … end
  end
end
```

Please, change the module (class) name *GeneralModuleTemplate* to the wished one (which should be the same as the module filename!).

Section zero contains the module constructor definition. Please, change the method name *GeneralModuleTemplate()* to the chosen module name. The single code line in this section will inherit the abstract module *Module* functionality and should be left as it is.

Section one contains pre-execution module routines, including universal for all modules *setUp()* method call from the abstract module *Module.* Implementation of this method in this particular

module could be filled in with the additional instructions, for example - folders creation for the output data and metadata as in the following code example:

```
function obj = setUp(obj)
        obj = setUp@Module(obj);

        % Create data_folder or metadata_folder in project folder
        createStandardFolder(obj.configuration, "data_folder", false);
        createStandardFolder(obj.configuration, "metadata_folder", false);
end
```

Section two is the main method *process()* for the module execution, which is called externally by the pipeline right after the *setUp()* method of the section one. The recommended structure of the method *process()* consists of three steps:

```
function obj = process(obj)
        %% Step 1. Get/set input data
        %% Step 2. Execute target module code
        %% Step 3. Link output data and metadata to standard locations
end
```

Step 1. Get/set input data. Put here code to get paths to the files and directories as well as configuration parameter values from the global configuration structure *obj.configuration* or calculate/set the necessary intermediate data as the fields of the temporal configuration structure *obj.dynamic_configuration*. The example code lines for this step are given below (more examples are available in the corresponding template file on GitHub):

```
        % Get global configuration parameter PARAM
        config_param = obj.configuration.PARAM;

        % Set dynamic configuration parameter PARAM with VALUE
        obj.dynamic_configuration.PARAM = VALUE;
```

Step 2. Execute target module code. Put here code to execute your data processing code. You might want to add a custom method(s) call(s):

```
        [OUTPUTS] = obj.customMethod(INPUTS);
```

which is defined in the optional section “N. Custom processing method” as the following:

```
function [outputs] = customMethod(obj, inputs)
        … // put here your code
end
```

You might also want to wrap external (CLI) software calls. In this case for all the available (data processing) execution methods (i.e. "once", "sequential", "parallel", "in_order") you may use the following command call:

```
executeCommand(obj.configuration.tool_command...
        + " -arg1 " + num2str(argument_value_number)...
        + " -arg2 " + strjoin(num2str(argument_value_numbers_list), ",")...
        ... % some other parameters
        + " -argN " + argument_value_string, false, obj.log_file_id);
```

where *obj.configuration.tool_command* is the parameter for the external command call (for example, binary file name or file path), *obj.log_file_id* is the log file identifier to write the command output to, and *argument_X* are examples of parameters of different types to be passed to the command call.

If you want to code module to be parallelized on CPUs using "parallel" execution method or on GPUs using "in_order" execution method, the assigned index of a parallelized piece of data (currently only tilt serie/tomogram, but can be else) to be processed on the assigned CPU or GPU worker is accessible by calling *obj.configuration.set_up.adjusted_j*.

To access the PARAM parameter of the corresponding computing resource-assigned tomogram or tilt serie use:

```
data =
obj.configuration.tomograms.(field_names{obj.configuration.set_up.adjusted_j}).PARAM;
```

Additionally, for GPU-parallelized module execution (when *execution_method* parameter is set to "in_order") to get assigned GPU use:

```
if obj.configuration.set_up.gpu > 0
    % TomoBEAR uses “1” as the first index convention for GPU IDs
    gpu_number = obj.configuration.set_up.gpu - 1;
end
```

<u>Step 3. Link output data and metadata to standard locations.</u> Put here code to make symbolic links to the intermediate or final output (meta)data:

- using source file and destination file paths:
  createSymbolicLink(source_file_path, dest_file_path, obj.log_file_id);

- using source file and destination directory paths where destination dir is a “(meta)data_folder/” in project directory:
  createSymbolicLinkInStandardFolder(obj.configuration, source_file_path, "data_folder", obj.log_file_id);

For each of the described above steps as a good software engineering practice we strongly recommend to add necessary execution messages in order to inform user about execution status by INFO and ERROR messages using example commands below:

```
% Display info-message for user during execution
disp("INFO: Put some info message here...");

% Display error message for user during execution
error("ERROR: Put some error message here!");
```

Section three contains post-execution module routines, including universal for all modules *cleanUp()* method calls from the abstract module *Module*. Implementation of this method in this particular module could be filled in with the additional instructions, for example - intermediate (meta)data folders or files deletion as in the following code example:

```
function obj = cleanUp(obj)
        % Automated data removal (execution modes except "cleanup")
        obj.deleteFilesOrFolders(files); % for files with folders
        obj.deleteFolderIfEmpty(folder); % for folders only

        % Automated data removal ("cleanup" execution mode only)
        if obj.configuration.execute == false && obj.configuration.keep_intermediates == false
           % put here your code for cleanup
        end

        % Post-execution routines (execution modes except "cleanup")
        obj = cleanUp@Module(obj);
end
```

All the sections, except for optional section N, are mandatory and their names have to be left as in the presented template file.

Further notes

The second module template is oriented on wrapping an external Python-based deep learning-tool, which contains a typical structure consisting of pre-processing, training, prediction and post-processing stages. The commented code of the corresponding module template is publicly available on the TomoBEAR GitHub repository, as well as some additional developer-level details and the repository contribution guidelines.

Project and source code structure

Here we additionally provide a description of the TomoBEAR project and source code folders.

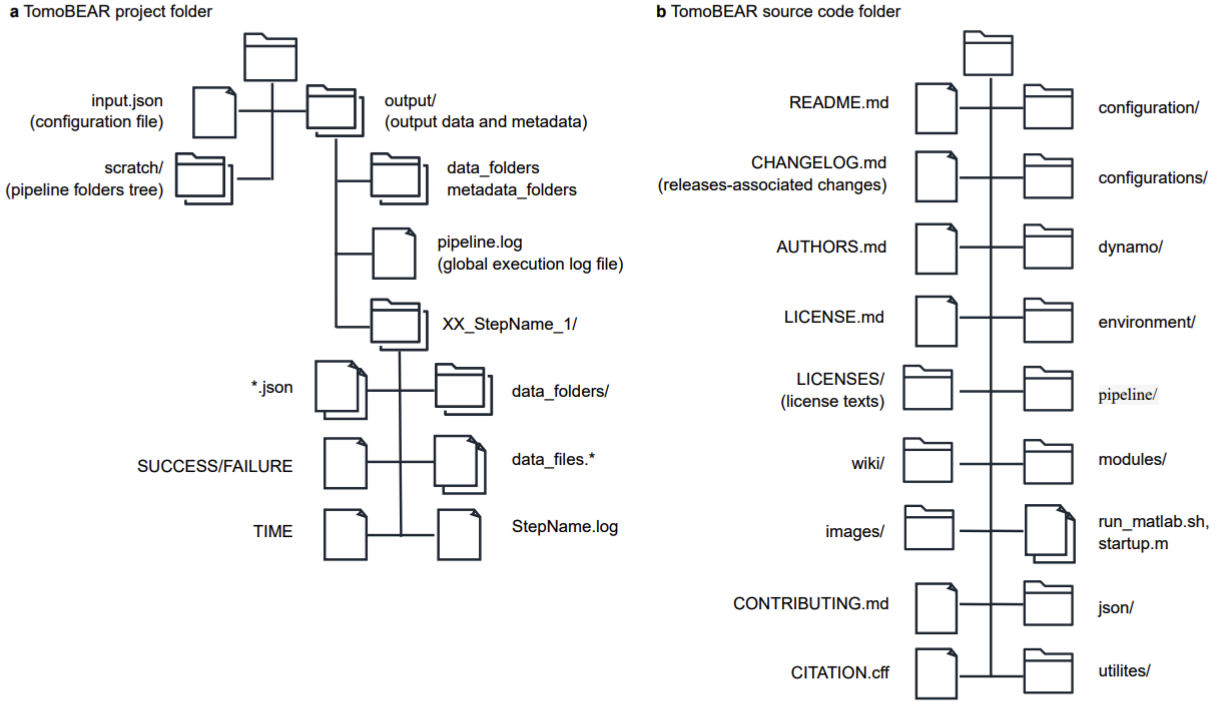

TomoBEAR project folder is structured as presented on the figure above (panel A) with the following elements:

- "input.json" - the input JSON configuration file which users should create themselves;
- "output/" - the main output folder which contains all the kinds of data (input, intermediate, output) and metadata (for data and pipeline) structured in the following way:
    - "XX_StepName_1/" - those are processing steps folders, containing in general step-related "data_files.*" and "data_folders/" (for example, individual tomogram/tilt serie folders "tomogram_*/" for the modules like *CreateStacks*, *BinStacks*, *Reconstruct*), as well as step-related pipeline metadata files, like various JSON files ("input.json", "output.json", etc.), log file of the step execution "StepName.log", pipeline checkpoint file "SUCCESS" or "FAILURE" (depending on processing outcome) and execution time log file "TIME";
    - "pipeline.log" - general log file, tracking global pipeline execution messages (info/warnings/errors);
    - "data_folders/" and "metadata_folders/" - folders where the most important (meta)data is moved or linked to.
- "scratch/" - special folder which contains just the folder tree of the output/ folder reflecting workflow structure and serving as a reference during the pipeline execution.

TomoBEAR source code is structured as presented on the figure above (panel B) and consists of the following parts:

- **environment setup scripts/functions** (“run_matlab.sh”, “startup.m”, “environment/”) - those are for configuring environmental and MATLAB path variables;
- **processing modules** (“modules/”) - those are interface modules for TomoBEAR steps, wrapping external software and/or our developments functionality;
- **pipeline modules** (“pipeline/”) - those are pipeline execution modules for different computational environments;
- **modified external routines** (“dynamo/”) - special folder to merge main Dynamo source code with our re-implemented Dynamo routines during TomoBEAR configuration to virtually produce updated functional version of Dynamo;
- **auxiliary functionality and files** (“utilities/”, “configuration/”, “configurations/” and “json/”) - variety of scripts/functions as well as templates and sets of defaults, used elsewhere in the briefly described above main parts;

- **software metadata and documentation** (“wiki/”, “images/”, “README.md”, “CHANGELOG.md”, “CONTRIBUTING.md”, “AUTHORS.md”, “CITATION.cff”, “LICENSE.md”, “LICENSES/”) - users-oriented files describing how to configure, install and use TomoBEAR as well as developers-oriented files containing general information about TomoBEAR as an open-source project like authors and contributors list, licensing and citation information, list of changes by releases, contribution guidelines, etc.

Supplementary Text 3: A tutorial for structural determination of purified 80S ribosomes (EMPIAR 10064).

To provide an example of the TomoBEAR processing project we have chosen the well-known cryo-ET benchmarking data set containing 80S ribosomes - EMPIAR-10064.

In this tutorial we will:

- explain structure and important parameters of the input JSON file used as a configuration file in TomoBEAR projects;
- explain output data structure of TomoBEAR projects;
- explain pipeline execution and control by checkpoint files;
- guide you through the processing of the famous real-world data set EMPIAR-10064 to achieve resolution of 11.3 Å with 4003 particles in nearly-automated parallelized manner.

Video-tutorials

We have prepared a range of short (8-12 min) video-tutorials following the written version of the 80S ribosome tutorial provided below:

- Video-tutorial 0 (link: https://www.youtube.com/watch?v=2uizkE616tE): explaining how to get the latest TomoBEAR version and configure TomoBEAR and its dependencies;
- Video-tutorial 1 (link: https://www.youtube.com/watch?v=N93tfAXp990): description of the project configuration file and the pipeline execution;
- Video-tutorial 2 (link: https://www.youtube.com/watch?v=qbkRtMJp0eI): additional configuration file parameters description, TomoBEAR-IMOD-TomoBEAR loop for checking tilt series alignment results and fiducials refinement (if needed);
- Video-tutorial 3 (link: https://www.youtube.com/watch?v=BP2T_Y7BiDo): checking on further intermediate results (alignment, CTF-correction, reconstruction, template matching).

Step 0. Install and configure TomoBEAR

All the needed instructions for TomoBEAR installation and configuration you can find on TomoBEAR GitHub repository in documentation by the following address: https://github.com/KudryashevLab/TomoBEAR/wiki/Installation-and-Setup.

Step 1. Download tutorial data

You can download the EMPIAR-10064 data set by the following link (mixedCTEM part): https://www.ebi.ac.uk/empiar/EMPIAR-10064. After downloading the data, extract it in a folder of your choice.

In our case we used only the mixedCTEM data and achieved 11 Å in resolution with 4003 particles which is similar to the resolution achieved by the original researchers. If you want you can additionally use the CTEM data to be able to pick even more particles.

Step 2. Setup configuration file

In the TomoBEAR source code folder you will find a subfolder configurations/ containing a file ribosome_empiar_10064_dynamo.json. This file describes the processing pipeline which should be setup by TomoBEAR to process used in this tutorial data set.
The following paragraphs will explain the variables contained in the JSON file and the needed changes to be able to run TomoBEAR on your local machine.
Firstly and most importantly you need to show TomoBEAR the path to the data and the processing folder. This must be done in the section "general": {} of the JSON file, as shown below:

```
   "general": {
        "project_name": "Ribosome",
        "project_description": "Ribosome EMPIAR 10064",
        "data_path": "/path/to/the/ribosome/data/*.mrc",
        "processing_path": "/path/to/the/processing/folder/",
        "expected_symmetrie": "C1",
        "apix": 2.62,
        "tilt_angles": [-60.0, -58.0, -56.0, -54.0, -52.0, -50.0, -48.0, -46.0,
-44.0, -42.0, -40.0, -38.0, -36.0, -34.0, -32.0, -30.0, -28.0, -26.0, -24.0, -
22.0, -20.0, -18.0, -16.0, -14.0, -12.0, -10.0, -8.0, -6.0, -4.0, -2.0, 0.0,
2.0, 4.0, 6.0, 8.0, 10.0, 12.0, 14.0, 16.0, 18.0, 20.0, 22.0, 24.0, 26.0, 28.0,
30.0, 32.0, 34.0, 36.0, 38.0, 40.0, 42.0, 44.0, 46.0, 48.0, 50.0, 52.0, 54.0,
56.0],
        "rotation_tilt_axis":-5,
        "gold_bead_size_in_nm": 9,
        "template_matching_binning": 8,
        "binnings": [2, 4, 8],
        "reconstruction_thickness": 1400,
        "as_boxes": false
  },
```

Since the pixel size and tilt angles are not provided in the header of MRC files of the data set EMPIAR-10064, we have to add this information in the "general": {} section of the configuration file.
Step 3. Execute until fiducials refinement

Everything else should be fine for now and the processing can be started. To run the TomoBEAR on the ribosome data set you need to open and pre-configure interactive MATLAB session by typing:
./run_matlab.sh

and execute the following command in the opened command window of MATLAB:
`runTomoBear("local", "/path/to/ribosome_empiar_10064_dynamo.json")`

or if you are using a compiled version of TomoBEAR and have everything set up properly, type in the following command on the command line from the TomoBEAR folder:./run_tomoBEAR local /path/to/ribosome_empiar_10064_dynamo.json /path/to/defaults.json. When you follow all the steps thoroughly, TomoBEAR should run up to the first appearance of StopPipeline. That means the following modules will be executed:

```
   "MetaData": {
    },
    "CreateStacks": {
    },
    "DynamoTiltSeriesAlignment": {
    },
    "DynamoCleanStacks": {
    },
    "BatchRunTomo": {
        "skip_steps": [4],
        "ending_step": 6
    },
    "StopPipeline": {
    },
```

This can take a while, as the result of this segment TomoBEAR will create a folder structure with subfolders for the individual steps. You can monitor the progress of the execution in shell and by inspecting the contents of the folders. Upon success of an operation, a file SUCCESS is written inside each folder. If you want to rerun a step you can terminate the process, change parameters, remove the SUCCESS file (or the entire subfolder) and restart the process.
Here the stacks have already been assembled, so neither "Motioncor2": {}, nor "SortFiles": {} modules were not needed. Here the key functionality is performed by "DynamoTiltSeriesAlignment": {} (the recommended tutorial: https://wiki.dynamo.biozentrum.unibas.ch/w/index.php/Walkthrough_on_GUI_based_tilt_series_alignment) after which the projections containing low number of tracked gold beads are excluded by "DynamoCleanStacks": {}. Finally, the output is converted into an IMOD project.
The running time depends on your infrastructure and setup.

Step 4. TomoBEAR-IMOD-TomoBEAR loop for fiducials refinement

After TomoBEAR stops you can inspect the fiducial model in the folder of "BatchRunTomo": {} which you can find in your processing folder.

```
cd /path/to/your/processing/folder/5_BatchRunTomo_1
```

Now you can inspect the alignment of every tilt stack one after the other and can possibly refine it if needed. For that you can use the following command. Please replace xxx with the tomogram number(s) that you want to inspect or * for all tomograms:etomo tomogram_xxx/*.edfWhen etomo starts - choose the fine alignment step which should be magenta-colored if everything went fine for that tomogram and then click on edit/view fiducial model to start 3dmod with

the right options to be able to refine the gold beads. If you do not see the green circles - please go to the IMOD command window, Edit -> Object l-> Type-> activate scattered and increase the size of the circle to e.g. 5.
Before you start to refine just press the arrow up button in the top left corner of the window with the viewport in order to zoom in. To refine the gold beads, click on `Go to the next big residual` in the window with the stacked buttons from top to bottom and the view in the viewport window should change to the location of a gold bead with a big residual.
Now see if you can center the marker better on the gold bead with the right mouse button. It is important that you do not put it on the peak of the red arrow, but center it on the gold bead. When you are finished with this gold bead press again on the `Go to next big residual` button. After you are finished with re-centering the marker on the gold beads you need to press the `Save and run tiltalign` button.

Step 5. Execute up to tomogram reconstructions and template matching

After you finish the inspection of all the alignments in all tomograms you can start `TomoBEAR` again as previously and it will continue from where it stopped up to the next `StopPipeline` section. To continue running `TomoBEAR` on the Ribosome data set you need to type in as previously the following command in the command window of MATLAB:
`runTomoBear("local", "/path/to/ribosome_empiar_10064_dynamo.json")`

or if you are using a compiled version of `TomoBEAR` and have everything set up properly type in the following command on the command line from the `TomoBEAR` folder:`./run_tomoBEAR local /path/to/ribosome_empiar_10064_dynamo.json /path/to/defaults.json`

`TomoBEAR` should now detect that it has stopped at the previous step `StopPipeline` and continue from where it stopped. The following excerpt from the `ribosome_empiar_10064_dynamo.json` file is describing what `TomoBEAR` needs to do next:

```
   "BatchRunTomo": {
        "starting_step": 8,
        "ending_step": 8
    },
    "GCTFCtfphaseflipCTFCorrection": {
    },
    "BatchRunTomo": {
        "starting_step": 10,
        "ending_step": 13
    },
    "BinStacks": {
    },
    "Reconstruct": {
    },
    "DynamoImportTomograms": {
    },
```

```
    "EMDTemplateGeneration": {
        "template_emd_number": "3420",
        "flip_handedness": true
    },
    "DynamoTemplateMatching": {
        "sampling": 15
        "size_of_chunk": [463, 463, 175]
    },
    "TemplateMatchingPostProcessing": {
        "cc_std": 2.5
    },
```

This segment performs estimation of defocus to calculate the **Contrast Transfer Function** (**CTF**) using Gctf and subsequent CTF-correction using Ctfphaseflip from IMOD (`"GCTFCtfphaseflipCTFCorrection": {}`). You can inspect the quality of fitting by going into the folder `8_GCTFCtfphaseflipCTFCorrection_1` and typing `imod tomogram_xxx/slices/*.ctf`and making sure that the Thon rings match the estimation. If not - play with the parameters of the `GCTFCtfphaseflipCTFCorrection` module.
Then binned aligned CTF-corrected stacks are produced by `"BinStacks": {}` and tomographic reconstructions are generated for the binnings specified in the section `"general": {}`. In this example the particles are picked using template matching. First a template from EMDB is produced at a proper voxel size, then `"DynamoTemplateMatching": {}` creates **cross-correlation** (**CC**) volumes which can be inspected. At a high binning level using the whole volume as a single chunk is more optimal than doing several chunks, so it is important to set the corresponding parameter to the size of the binned tomogram used for template matching. Finally, highest cross-correlation peaks, over 2.5 standard deviations above the mean value in the cross-correlation volume are selected for extraction to 3D particle files, the initial coordinates are stored in the `particles_table` folder as a file in the dynamo table format. You can inspect the cross-correlation volumes and set the threshold for more or less stringent extraction of particles.

Step 6. Execute subtomogram averaging (StA)

In the section below you will find **subtomogram classification projects** that should produce you a reasonable structure. They first use **multi-reference alignment projects** with a true class and so-called **"noise trap" classes** to first classify out false-positive particles produced by template matching; this happens at binning which was used for template matching. In the end of the segment you should have a reasonable set of particles in the best class.The example configuration section is the following:

```
    "DynamoAlignmentProject": {
        "iterations": 3,
        "classes": 4,
        "use_noise_classes": true,
        "use_symmetrie": false
    },
    "DynamoAlignmentProject": {
```

```
        "iterations": 3,
        "classes": 4,
        "use_noise_classes": true,
        "use_symmetrie": false,
        "selected_classes": [1]
    },
    "DynamoAlignmentProject": {
        "iterations": 3,
        "classes": 4,
        "use_noise_classes": true,
        "use_symmetrie": false,
        "selected_classes": [1]
    },
    "DynamoAlignmentProject": {
        "iterations": 3,
        "classes": 4,
        "use_noise_classes": true,
        "use_symmetrie": false,
        "selected_classes": [1]
    },
    "DynamoAlignmentProject": {
        "iterations": 3,
        "classes": 4,
        "use_noise_classes": true,
        "use_symmetrie": false,
        "selected_classes": [1]
    },
    "DynamoAlignmentProject": {
        "iterations": 3,
        "classes": 4,
        "use_noise_classes": true,
        "use_symmetrie": false,
        "selected_classes": [1]
    },
    "DynamoAlignmentProject": {
        "iterations": 3,
        "classes": 3,
        "use_noise_classes": true,
        "use_symmetrie": false,
        "selected_classes": [1],
        "box_size": 1.10,
        "binning": 4
    },
```

```
    "StopPipeline": {
    },
```

After subtomogram classification the projects are done, you should have a reasonable set of particles in the best class which you should select. To select the best class, you need to go into the last `DynamoAlignmentProject` folder before the last produced `StopPipeline` folder, and then go to
`alignment_project_1_bin_y/mraProject_bin_y/results/iteQQQQ/averages/` (where `iteQQQQ` corresponds to the pre-last iteration folder) and type
`imod average_ref_CCC_ite_QQQQ.em`

to open produced average for each class `CCC` to identify the best class to use further.
Such variables as binning `y`, pre-last iteration number `QQQQ` and class numbers `CCC` can depend on parameters used in `DynamoAlignmentProject`. But if you repeat instructions provided in this tutorial, this should be the folder:
`23_DynamoAlignmentProject_1/alignment_project_1_bin_4/mraProject_bin_4/results/ite0012/averages/` where you may find files `average_ref_001_ite_0012.em`, `average_ref_002_ite_0012.em`, and `average_ref_003_ite_0012.em` corresponding to the produced averages for 3 classes, from which you should choose the best one.
Once you have selected the best class, insert corresponding class number in the list `[]` as a value of the parameter `"selected_classes"` to the following section to be executed by `TomoBEAR`:

```
   "DynamoAlignmentProject": {
        "classes": 1,
        "iterations": 1,
        "use_noise_classes": false,
        "swap_particles": false,
        "use_symmetrie": false,
        "selected_classes": [3],
        "binning": 4,
        "threshold":0.8
    },
```

The section above is called a **single reference project**, which will split the particles of the previously selected best class into two equally sized classes (called even/odd halves) with subsequent alignment of the particles in those halves to produce corresponding averages. This division will be needed further when unbinned data will be produced to be able to calculate the resolution of the resulting averaged map using **Fourier Shell Correlation** (**FSC**) curve.
After the first single reference project introduced above you will need to process tomograms by similar projects but at lower binnings in order to reduce the voxel size up to unbinned data to get the information corresponding to the highest possible resolution to be achieved using the current data set. At this point automated workflow is finished as the user needs to play with the masks, particle sets, etc.
You may want to try to use the following example of the end section of `JSON` file in order to have experience of processing tomograms at lower binnings to produce unbinned data to finally be able

to calculate resolution of your ribosome density map as a result of the first experience with `TomoBEAR`:

```
   "DynamoAlignmentProject": {
        "classes": 1,
        "iterations": 1,
        "use_noise_classes": false,
        "swap_particles": false,
        "use_symmetrie": false,
        "selected_classes": [1,2],
        "binning": 2,
        "threshold":0.9
    },
    "BinStacks":{
        "binnings": [1],
        "use_ctf_corrected_aligned_stack": false,
        "run_ctf_phaseflip": true
    },
    "Reconstruct": {
        "reconstruct": "unbinned"
    },
    "DynamoAlignmentProject": {
        "classes": 1,
        "iterations": 1,
        "use_noise_classes": false,
        "swap_particles": false,
        "use_symmetrie": false,
        "selected_classes": [1,2],
        "binning": 1,
        "threshold":1
    }
```

Consider, that after performing first single reference project you need to select both halves from the previous step by setting `"selected_classes": [1,2]` (in order to keep all particles) while producing one class by setting `"classes": 1` at the subsequent steps of binning reduction using `"DynamoAlignmentProject": {}` module.

If you get `out of memory` error while running some of `"DynamoAlignmentProject": {}` at lower binnings (especially the last one), you may put additional parameter `"dt_crop_in_memory": 0` to the corresponding `"DynamoAlignmentProject": {}` sections in order to prevent keeping the whole tomogram in memory for processing. For example, in this tutorial the size of the one of unbinned tomograms is ~72Gb, while for binning 2 it is near 9Gb.

Step 7. Estimate resolution of the output final map

Finally, to estimate resolution of produced by `TomoBEAR` results, you need to use the following `Dynamo` command in `MATLAB`:

```
fsc = dfsc(path_to_half1, path_to_half2, 'apix', 2.62, 'mask', path_to_mask,
'show', 'on')
```

where `path_to_half1` and `path_to_half2` are paths to the pre-last iteration results of the last `DynamoAlignmentProject` folder, which in this tutorial are located in `29_DynamoAlignmentProject_1/alignment_project_1_bin_1/mraProject_bin_1_eo/results/ite0006/averages`, where you may find files `average_ref_001_ite_0006.em` and `average_ref_002_ite_0006.em` corresponding to the averages made from halves of the resulting particles set. You also need to use a mask to filter averages for FSC calculation, and the accuracy of the used mask has an impact on the resolution estimation. Appropriate mask to use for the initial resolution estimation you may find in the last `DynamoAlignmentProject` folder in a file called `mask.em` (in this tutorial `path_to_mask` is `29_DynamoAlignmentProject_1/mask.em`).

After that you should get a similar FSC curve to the following one:

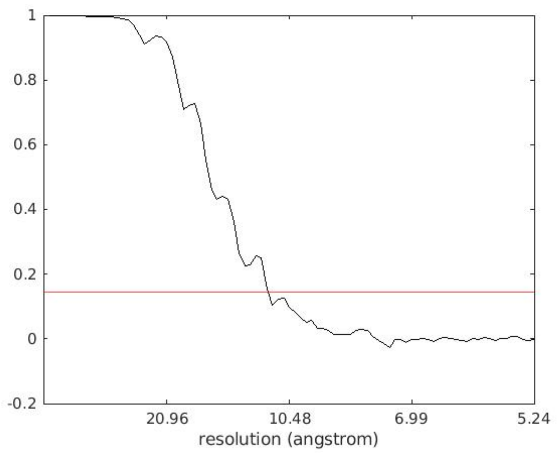

where in red we added the `FSC at 0.143` threshold to estimate the global resolution of the final map, which in our case for the final set of 4003 ribosome particles reached 11 Å.

Supplementary Text 4: Mini-guide on live data processing

The “live” processing mode is a TomoBEAR feature which allows processing of data “on-the-fly”, i.e. as it comes from the microscope. The main purpose of this mode is to screen sample quality, for example to identify presence of the molecular target in the sample. Main prerequisite for “live” mode is availability of TomoBEAR access to the data folder, where the collected files arrive.

The “live” mode is available for dose-fractionated movies in TIF or MRC format, recorded using single-shot data collection. During this type of data record the collected tilt series are processed sequentially in their arrival order.

To properly configure configuration input JSON file for “live” mode, the user needs to set the following two parameters in the “general” section:

- “minimum_files” - number of files needed to consider tilt serie to be fully collected and subjected to processing (default: 15);
- “Listening_time_threshold_in_minutes” - threshold for a period of time passed from the latest arrived file of a tilt serie upon which that tilt serie would be considered as a fully collected and subjected to processing regardless to the number of collected files (default: 15 [min]).

To reduce the processing time in the “live” mode we implemented the option of a simple summation of dose-fractionated movies into corresponding tilt images instead of the full motion correction procedure. As well, the user may skip computationally expensive CTF estimation and correction steps. Corresponding example of the suggested JSON file for live data processing setup is accessible by the source code folder path “configurations/live_template.json” and is provided below:

```
{
  "general": {
    "project_name": "your_project_name",
    "project_description": "your project name description",
    "data_path": "/path/to/live/data/folder/prefix*.tif",
    "processing_path": "/path/to/processing/folder",
    "expected_symmetrie": "Cx",
    "gold_bead_size_in_nm": xx,
    "rotation_tilt_axis": xx,
    "aligned_stack_binning": 8,
    "pre_aligned_stack_binning": 8,
    "reconstruction_thickness": xxxx,
    "as_boxes": false,
    "minimum_files": 41,
    "ft_bin": 2,
    "listening_time_threshold_in_minutes": 10
  },
  "MetaData": {
```

```
    },
    "SortFiles": {
    },
    "MotionCor2": {
        "method": "SumOnly",
        "execution_method": "sequential"
    },
    "CreateStacks": {
    },
    "DynamoTiltSeriesAlignment": {
        "use_newstack_for_binning": true
    },
    "DynamoCleanStacks": {
    },
    "BatchRunTomo": {
        "skip_steps": [4, 7, 9],
        "ending_step": 13
    },
    "BinStacks": {
    },
    "Reconstruct": {
        "generate_exact_filtered_tomograms": true,
        "exact_filter_size": xxxx
    }
}
```

Since “live” data processing mainly serves for sample quality check, in order to additionally reduce processing time - we advise to use the following setup (as in the example provided above):

- set "ft_bin" parameter value to at least 2 in order to pre-bin views (summarized/motion-corrected dose-fractionated movies) of pre-processed stack for all subsequent modules;
- use high binning values for "pre_aligned_stack_binning" and "aligned_stack_binning" parameters (for example, 8 as above);
- enable "use_newstack_for_binning" by setting it to “true” in “DynamoTiltSeriesAlignment” step;

In order to improve contrast in reconstructions we would recommend enabling "generate_exact_filtered_tomograms" and setting up "exact_filter_size" to define filters to be used to produce contrast-enhanced reconstructions.

If you start data collection from zero-tilt, to avoid problems of files perception and sorting caused by -0.0/+0.0 appearing as the angle for non-tilted views (due to small initial tilting offset being present) instead of expected 0.0 it is also recommended to add "first_tilt_angle": 0 to the “general” section of your input JSON file. The same parameter is useful as well when the sample was pre-tilted (for example, for in situ FIB-milled sample) to explicitly set the pre-tilt angle value to be used.

Finally, to execute the workflow, the user needs to start MATLAB interactive session from the cloned TomoBEAR source code folder by

./run_matlab.sh

and launch the TomoBEAR in “live” mode by typing in the MATLAB command window the following command:

runTomoBear("local_live", "/path/to/input.json", "/path/to/defaults.json")

REFERENCES FOR SUPPLEMENTARY MATERIAL:

1. Zheng, S. Q. *et al.* MotionCor2: anisotropic correction of beam-induced motion for improved cryo-electron microscopy. *Nat. Methods* **14**, 331–332 (2017).
2. Sanchez, R. M., Zhang, Y., Chen, W., Dietrich, L. & Kudryashev, M. Subnanometer-resolution structure determination in situ by hybrid subtomogram averaging - single particle cryo-EM. *Nat. Commun.* **11**, 3709 (2020).
3. Castaño-Díez, D., Kudryashev, M., Arheit, M. & Stahlberg, H. Dynamo: a flexible, user-friendly development tool for subtomogram averaging of cryo-EM data in high-performance computing environments. *J. Struct. Biol.* **178**, 139–151 (2012).
4. Zheng, S. *et al.* AreTomo: An integrated software package for automated marker-free, motion-corrected cryo-electron tomographic alignment and reconstruction. *J. Struct. Biol. X* **6**, 100068 (2022).
5. Mastronarde, D. N. & Held, S. R. Automated tilt series alignment and tomographic reconstruction in IMOD. *J. Struct. Biol.* **197**, 102–113 (2017).
6. Zhang, K. Gctf: Real-time CTF determination and correction. *J. Struct. Biol.* **193**, 1–12 (2016).
7. Rohou, A. & Grigorieff, N. CTFFIND4: Fast and accurate defocus estimation from electron micrographs. *J. Struct. Biol.* **192**, 216–221 (2015).
8. Liu, Y.-T. *et al.* Isotropic reconstruction for electron tomography with deep learning. *Nat. Commun.* **13**, 6482 (2022).
9. Castaño-Díez, D., Kudryashev, M. & Stahlberg, H. Dynamo Catalogue: Geometrical tools and data management for particle picking in subtomogram averaging of cryo-electron tomograms. *J. Struct. Biol,* **197**, 135-144 (2016)
10. Wagner, T. *et al.* SPHIRE-crYOLO is a fast and accurate fully automated particle picker for cryo-EM. *Commun. Biol.* **2**, 1–13 (2019).
