# Supplementary material for: Streamlined structure determination by cryo-electron tomography and subtomogram averaging using TomoBEAR
Source: Nat Commun. 2023 Oct 17;14:6543. doi: 10.1038/s41467-023-42085-w (PMC10582028; doi:10.1038/s41467-023-42085-w)
Supplement: Supplementary file 3 — Reporting Summary [file 41467_2023_42085_MOESM3_ESM.pdf]

nature portfolio

Corresponding author(s): MIkhail Kudryashev

Last updated by author(s): 26/9/23

Reporting Summary

Nature Portfolio wishes to improve the reproducibility of the work that we publish. This form provides structure for consistency and transparency in reporting. For further information on Nature Portfolio policies, see our Editorial Policies and the Editorial Policy Checklist.

Statistics

For all statistical analyses, confirm that the following items are present in the figure legend, table legend, main text, or Methods section.

n/a	Confirmed	
☒	☐	The exact sample size (*n*) for each experimental group/condition, given as a discrete number and unit of measurement
☒	☐	A statement on whether measurements were taken from distinct samples or whether the same sample was measured repeatedly
☒	☐	The statistical test(s) used AND whether they are one- or two-sided<br>*Only common tests should be described solely by name; describe more complex techniques in the Methods section.*
☒	☐	A description of all covariates tested
☒	☐	A description of any assumptions or corrections, such as tests of normality and adjustment for multiple comparisons
☒	☐	A full description of the statistical parameters including central tendency (e.g. means) or other basic estimates (e.g. regression coefficient) AND variation (e.g. standard deviation) or associated estimates of uncertainty (e.g. confidence intervals)
☒	☐	For null hypothesis testing, the test statistic (e.g. *F*, *t*, *r*) with confidence intervals, effect sizes, degrees of freedom and *P* value noted<br>*Give P values as exact values whenever suitable.*
☒	☐	For Bayesian analysis, information on the choice of priors and Markov chain Monte Carlo settings
☒	☐	For hierarchical and complex designs, identification of the appropriate level for tests and full reporting of outcomes
☒	☐	Estimates of effect sizes (e.g. Cohen's *d*, Pearson's *r*), indicating how they were calculated

Our web collection on statistics for biologists contains articles on many of the points above.

Software and code

Policy information about availability of computer code

Data collection	EMPIAR database: raw data as data sets for the benchmark;<br>EMDB database: subtomogram average templates for template matching;<br>Digital Micrograph-3.44.3447.0 (Gatan);<br>SerialEM-4.0.0beta (https://doi.org/10.1017/S1431927603445911);<br>Titan Server-3.12.1 (Thermo Fisher Scientific).

Data analysis	MATLAB-2021a/b (MathWorks); CUDA-10.1/11.5 (freeware, NVIDIA); Anaconda-4.14.0 (freeware, Continuum analytics); TensorFlow 1.15 (https://www.tensorflow.org/); Python-3.8/3.9 (https://www.python.org/) IMOD-4.11.24/4.9.12 (doi:10.1006/jsbi.1996.0013; doi:10.1016/j.jsb.2016.07.011); Dynamo-1.1.532 (doi:10.1016/j.jsb.2011.12.017) and its Dynamo Catalogue system (doi:10.1016/j.jsb.2016.06.005); MotionCor2-1.4.4 (doi:10.1038/nmeth.4193); AreTomo-1.3.3 (doi:10.1016/j.yjsbx.2022.100068); GCTF-v1.18 (doi:10.1016/j.jsb.2015.11.003); CTFFIND-4.1.14 (doi:10.1016/j.jsb.2015.08.008); IsoNet-0.2 (doi:10.1038/s41467-022-33957-8); crYOLO-1.9.3 (doi:10.1038/s42003-019-0437-z); RELION-4.0 (doi:10.1101/2022.02.28.482229); SUSAN-v0.1-RC1-TomoBEAR (doi:10.5281/zenodo.7950904); TomoBEAR-v0.4.0 (doi: 10.5281/zenodo.8221247); USCF ChimeraX-1.5 (https://www.rbvi.ucsf.edu/chimerax).

For manuscripts utilizing custom algorithms or software that are central to the research but not yet described in published literature, software must be made available to editors and reviewers. We strongly encourage code deposition in a community repository (e.g. GitHub). See the Nature Portfolio guidelines for submitting code & software for further information.

Data

Policy information about availability of data

All manuscripts must include a data availability statement. This statement should provide the following information, where applicable:

- Accession codes, unique identifiers, or web links for publicly available datasets
- A description of any restrictions on data availability
- For clinical datasets or third party data, please ensure that the statement adheres to our policy

TomoBEAR is open-source, available on GitHub https://github.com/KudryashevLab/TomoBEAR; the 80S ribosome data set and corresponding input preset files are available as a tutorial. For the apoferritin data set, the raw microscope data was deposited in the Electron Microscopy Public Image Archive (EMPIAR) with the accession code EMPIAR-11543. The corresponding final subtomogram averages are available in the Electron Microscopy Data Bank (EMDB) under the deposition codes EMD-17232 (apoferritin), EMD-17272 (RyR1).The 80S ribosome from the plasmaFIB dataset will be deposited to EMDB and the accession code will be stated here.

Research involving human participants, their data, or biological material

Policy information about studies with human participants or human data. See also policy information about sex, gender (identity/presentation), and sexual orientation and race, ethnicity and racism.

Reporting on sex and gender	na
Reporting on race, ethnicity, or other socially relevant groupings	na
Population characteristics	na
Recruitment	na
Ethics oversight	na

Note that full information on the approval of the study protocol must also be provided in the manuscript.

Field-specific reporting

Please select the one below that is the best fit for your research. If you are not sure, read the appropriate sections before making your selection.

☒ Life sciences ☐ Behavioural & social sciences ☐ Ecological, evolutionary & environmental sciences

For a reference copy of the document with all sections, see nature.com/documents/nr-reporting-summary-flat.pdf

Life sciences study design

All studies must disclose on these points even when the disclosure is negative.

Sample size	we picked all the particles in tomograms that had highest cross correlation values, limiting to 1000 particles per tomogram. The details are described in the results/methods sections
Data exclusions	We performed multi reference classifications and kept the good classes. The details are described in the results/methods sections

Replication	the data for this research is publically available and we are depositing our structures.
Randomization	random distribution to groups as performed in Relion4.0 or in Dynamo (for the purified ribosome dataset)
Blinding	na

Reporting for specific materials, systems and methods

We require information from authors about some types of materials, experimental systems and methods used in many studies. Here, indicate whether each material, system or method listed is relevant to your study. If you are not sure if a list item applies to your research, read the appropriate section before selecting a response.

Materials & experimental systems

n/a	Involved in the study
☒	☐ Antibodies
☒	☐ Eukaryotic cell lines
☒	☐ Palaeontology and archaeology
☒	☐ Animals and other organisms
☒	☐ Clinical data
☒	☐ Dual use research of concern
☒	☐ Plants

Methods

n/a	Involved in the study
☒	☐ ChIP-seq
☒	☐ Flow cytometry
☒	☐ MRI-based neuroimaging
